# Intratumor heterogeneity of *HMCN1* mutant alleles associated with poor prognosis in patients with breast cancer

**DOI:** 10.18632/oncotarget.26071

**Published:** 2018-09-07

**Authors:** Chie Kikutake, Minako Yoshihara, Tetsuya Sato, Daisuke Saito, Mikita Suyama

**Affiliations:** ^1^ Medical Institute of Bioregulation, Kyushu University, Fukuoka 812-8582, Japan; ^2^ AMED-CREST, Japan Agency for Medical Research and Development, Fukuoka 812-8582, Japan

**Keywords:** breast cancer, variant allele frequency, lymph node metastasis, genetic variant, next-generation sequencing

## Abstract

Human breast cancers comprise a complex and highly heterogeneous population of tumor cells. Intratumor heterogeneity is an underlying cause of resistance to effective therapies and disease recurrence. To explore prognostic factors based on intratumor heterogeneity, we analyzed genomic mutations in breast cancer patients registered in The Cancer Genome Atlas. We calculated the variant allele frequency (VAF) at each mutation site and evaluated the associations of VAFs with the prognosis of breast cancer. VAFs of *HMCN1* correlated with the prognosis and lymph node status. Although the detailed function of HMCN1 remains unknown, it is located in extracellular matrix and the mutation in the gene might be associated with cancer cell invasion and metastasis. This finding suggests that *HMCN1* is a potential metastatic factor and can be a candidate gene for targeted breast cancer therapy.

## INTRODUCTION

Breast cancer is the most common type of cancer affecting women worldwide. In 2012, approximately 1.7 million cases of breast cancer were newly diagnosed [1]. Changes in dietary habits and a reduced birth rate can increase the risk of breast cancer. Breast cancer is a clinically heterogeneous disease for which four basic therapeutic or molecular subtypes have been classified based on the expression status of three receptors: estrogen receptor, progesterone receptor, and human epidermal growth factor receptor 2 [2, 3]. Immunohistochemistry is used to classify these four tumor subtypes and ensure that effective treatment is provided to each patient.

Despite recent therapeutic advances, tumor recurrence and drug resistance remain major challenges in the field of breast cancer. These challenges are mainly attributed to intratumor heterogeneity [4], which is characterized by subclonal diversity within a tumor that originates from the accumulation of various somatic mutations during cell division and proliferation [5–7]. Intratumor heterogeneity has already been identified in several types of cancer, including breast, prostate, kidney, brain, liver, and lung cancers [8]. Drug-resistant subclones may develop via clonal evolution and reside at low frequencies within a tumor; after drug therapy, however, these subclones become the main population, leading to recurrence [9–11].

Intratumor heterogeneity can be most directly evaluated from DNA sequences using next-generation sequencing (NGS). One of the commonly used methods to analyze heterogeneity is the sequencing of samples from multiple regions of the same tumor [10, 12]. Ultra-deep sequencing can also be used to detect mutations with extremely low allele frequencies. Variant allele frequency (VAF), calculated as the proportion of reads with mutations at the variant site, is used as an index of heterogeneity [13, 14]. VAFs in a tumor can be used to determine the cellular prevalence of a mutation within a sample and estimate subpopulation frequencies and the tumor evolutionary process [15–17]. For example, deep sequencing was used to evaluate mutational processes of 21 breast cancers, leading to the finding that every tumor harbored a distinct subclonal lineage [10]. The use of NGS and analytical methods to define clonal heterogeneity has also provided insights into the genetic processes underlying breast cancer metastasis [18]. Recent studies also showed that clonal distribution based on VAF correlated with prognosis [19, 20]. Additionally, heterogeneity can be evaluated using large datasets generated by The Cancer Genome Atlas (TCGA) or the International Cancer Genome Consortium [21, 22]. Despite these advances, it remains highly challenging to identify tumor genetic factors associated with tumor growth or metastasis, as tumors exhibit considerable heterogeneity.

Several recent studies based on TCGA data have focused on the identification of driver genes and identified pathways containing potential drug targets [23]. These studies have accelerated the development of pathway-specific inhibitory drugs. Although mutations in breast cancer driver genes such as *TP53*, *PIK3CA*, and *GATA3* have been extensively investigated, somatic alterations in other genes are also believed to be associated with breast cancer [24]. To gain better insights into the extent of intratumor heterogeneity, we analyzed breast cancer genome sequencing data from TCGA. In this study, we focused on genes associated with breast cancer prognosis.

## RESULTS

### Identification of genes with high frequencies of mutations

**Figure 1:**
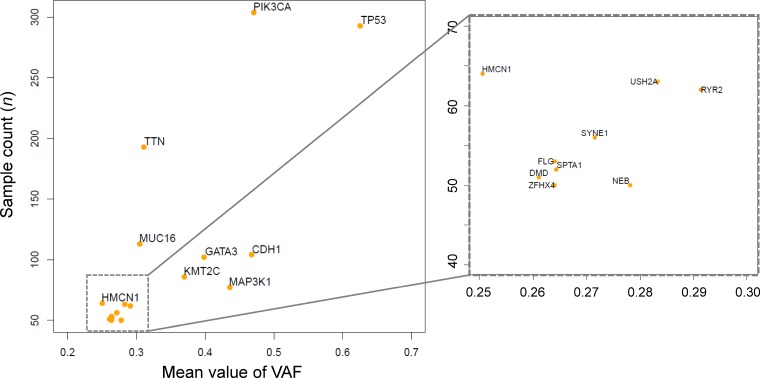
Frequently mutated genes and mean variant allele frequencies (VAFs) The scatter plot depicts 17 genes harboring mutations in > 50 samples. The x-axis indicates the mean VAF, and the y-axis indicates the number of samples with mutations. The plot at right is an enlargement of the square area enclosed by dotted lines in the left plot.

We sought genes with one of four types of mutation (missense mutations, nonsense mutations, frameshift insertions, and frameshift deletions) in ≥ 50 samples derived from the 1,044 breast cancer datasets in TCGA. We identified 17 such genes (Table 1) and calculated the mean VAF for each in the sample containing mutations (Figure 1). All VAFs were adjusted for tumor purity taken from the previous study [25]. The mean VAFs of already known driver genes in breast cancer, such as *TP53*, *PIK3CA*, and *CDH1* were found to be relatively high.

**Table 1:** Frequently mutated genes and mean variant allele frequencies and hazard ratios

Gene	Sample count	Mean value of VAF	Hazard Ratio (95% CI)	*P*-value	FDR^a^
*PIK3CA*	304	0.471	1.78 (0.729–4.348)	0.206	0.696
*TP53*	293	0.626	1.276 (0.455–3.581)	0.643	0.994
*TTN*	193	0.311	1.85 (0.843–4.06)	0.125	0.531
*MUC16*	113	0.305	1.768 (0.619–5.048)	0.287	0.696
*CDH1*	104	0.468	1.019 (0.263–3.951)	0.979	0.999
*GATA3*	102	0.398	0.859 (0.245–3.01)	0.813	0.999
*KMT2C*	86	0.370	1.327 (0.478–3.687)	0.587	0.994
*MAP3K1*	77	0.436	0.114 (0.013–0.985)	0.048	0.287
*HMCN1*	64	0.251	11.441 (2.065–63.406)	0.005	0.090^*^
*USH2A*	63	0.283	1.185 (0.245–5.74)	0.833	0.999
*RYR2*	62	0.291	0.059 (0.003–1.008)	0.051	0.287
*SYNE1*	56	0.272	1.635 (0.11–24.181)	0.721	0.999
*FLG*	53	0.264	0.342 (0.04–2.923)	0.327	0.696
*SPTA1*	52	0.264	1.965 (0.531–7.274)	0.312	0.696
*DMD*	51	0.261	1.87 (0.396–8.831)	0.429	0.811
*NEB*	50	0.278	1.124 (0.135–9.342)	0.914	0.999
*ZFHX4*	50	0.264	-	-	-

Abbreviations: 95% CI, 95% confidence interval; VAF, variant allele frequency; FDR, false discovery rate.

^a^Astarisk indicates FDR < 0.1.

^b^*ZFHX4* VAF could not be analyzed because sample size is small.

To examine the association of overall survival (OS) with VAFs of these 17 genes, we applied a Cox proportional hazards regression analysis with the covariates of age, tumor grade, and VAFs. In this analysis, the samples were divided into two groups using a VAF of 0.30 (i.e., 30%) as a cutoff. We used this cutoff because a previous study, which focused on samples with high tumor purity (≥ 70%), considered that a VAF of ≥ 0.25 was more likely to be clonal, whereas lower values were more likely to be subclonal [20]. Assuming that average purity is 85% (range 70–100%) then the cutoff should be 0.3 (0.25/0.85 = 0.294). We conducted this analysis without adjusting for other covariates just for a screening of genes that are possibly associated with breast cancer prognosis. We corrected *P* values for multiple testing using Benjamini and Hochberg false discovery rate (FDR) [26]. VAFs of *HMCN1* was found to be possibly associated with breast cancer prognosis (FDR < 0.1) (Table 1), and we focused on *HMCN1*, for which no association with breast cancer has previously been reported.

**Figure 2:**
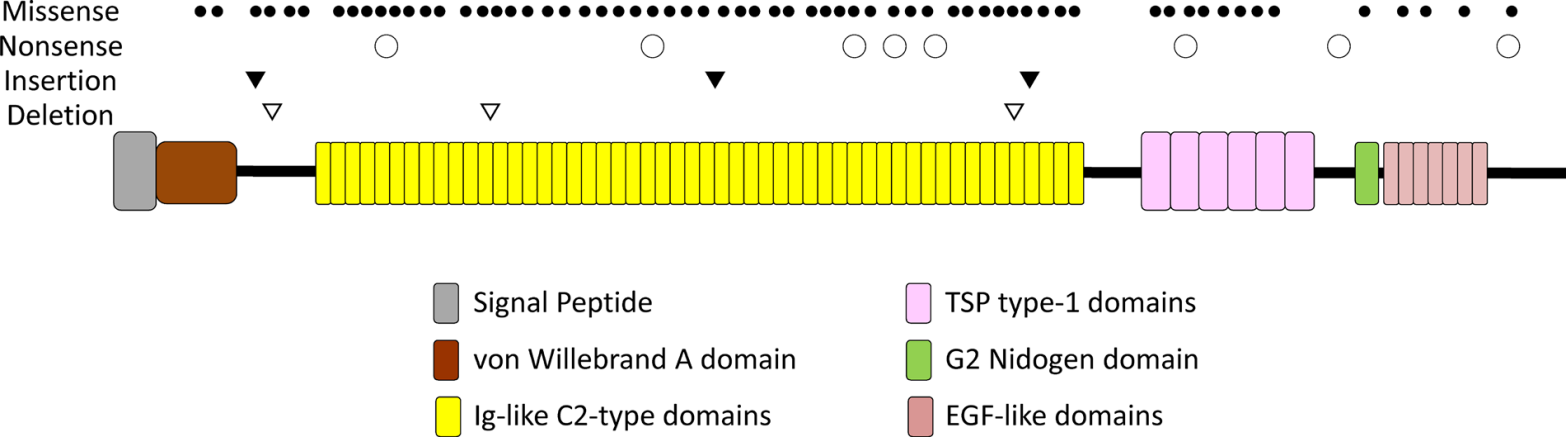
A schematic of the domains of human *HMCN1* (hemicentin-1) The types and positions of 78 somatic mutations are indicated above the diagram.

A total of 78 somatic mutations in *HMCN1* were detected in 6.1% (64/1,044) of samples (Table 2 and Figure 2). Among samples with detectable *HMCN1* mutations, 9.4% (6/64) contained two distinct mutations and 3.1% (2/64) contained more than two mutations. Of the 78 *HMCN1* mutations, 82.1% (64/78) were missense, whereas 10.3% (8/78) were nonsense and 7.7% (6/78) were indels. Furthermore, 69.2% (54/78) of the mutations were clustered in the Ig-like C2-type domains of HMCN1.

**Table 2:** Distribution of *HMCN1* mutations

HMCN1 Domains	Missense mutation	Nonsense mutation	Deletion	Insertion	Total
VWFA domain	2	0	0	0	2
Ig-like C2-type domains	45	5	2	2	54
TSP type-1 domains	8	1	0	0	9
Nidogen G2 beta-barrel domain	1	1	0	0	2
EGF-like domains	3	0	0	0	3
Other	5	1	1	1	8
Total	64	8	3	3	78

Abbreviations: EGF, epidermal growth factor; Ig, immunoglobulin; TSP, thrombospondins; VWFA, von Willebrand factor type A.

To evaluate the existence of any association between the mutation type and VAFs, we applied a one-way ANOVA to the data and found that the mean VAF values did not significantly differ among the four types of mutations (*P* = 0.430) (Supplementary Figure 1). We further evaluated the associations of the four molecular breast cancer subtypes with VAFs of *HMCN1*. However, an ANOVA indicated that the mean VAF values did not significantly differ among the four subtypes (*P* = 0.060) (Supplementary Figure 2).

### Expression of *HMCN1*

We compared the relative *HMCN1* mRNA expression levels among samples with higher (VAF of ≥ 0.30, *n* = 19) and lower VAFs (VAF of < 0.30, *n* = 45). As a result, we found that *HMCN1* expression levels did not significantly differ between the two groups (*P* = 0.343) (Supplementary Figure 3A). Additionally, we compared *TP53* and *PIK3CA* expression levels between the VAF groups and found no significant differences in either (*P* = 0.515 and 0.300, respectively) (Supplementary Figure 3B and 3C). We also found no significant differences in the relative *HMCN1* mRNA expression levels between *HMCN1* mutant and wild-type samples (*P* = 0.984) (Supplementary Figure 3D).

**Figure 3:**
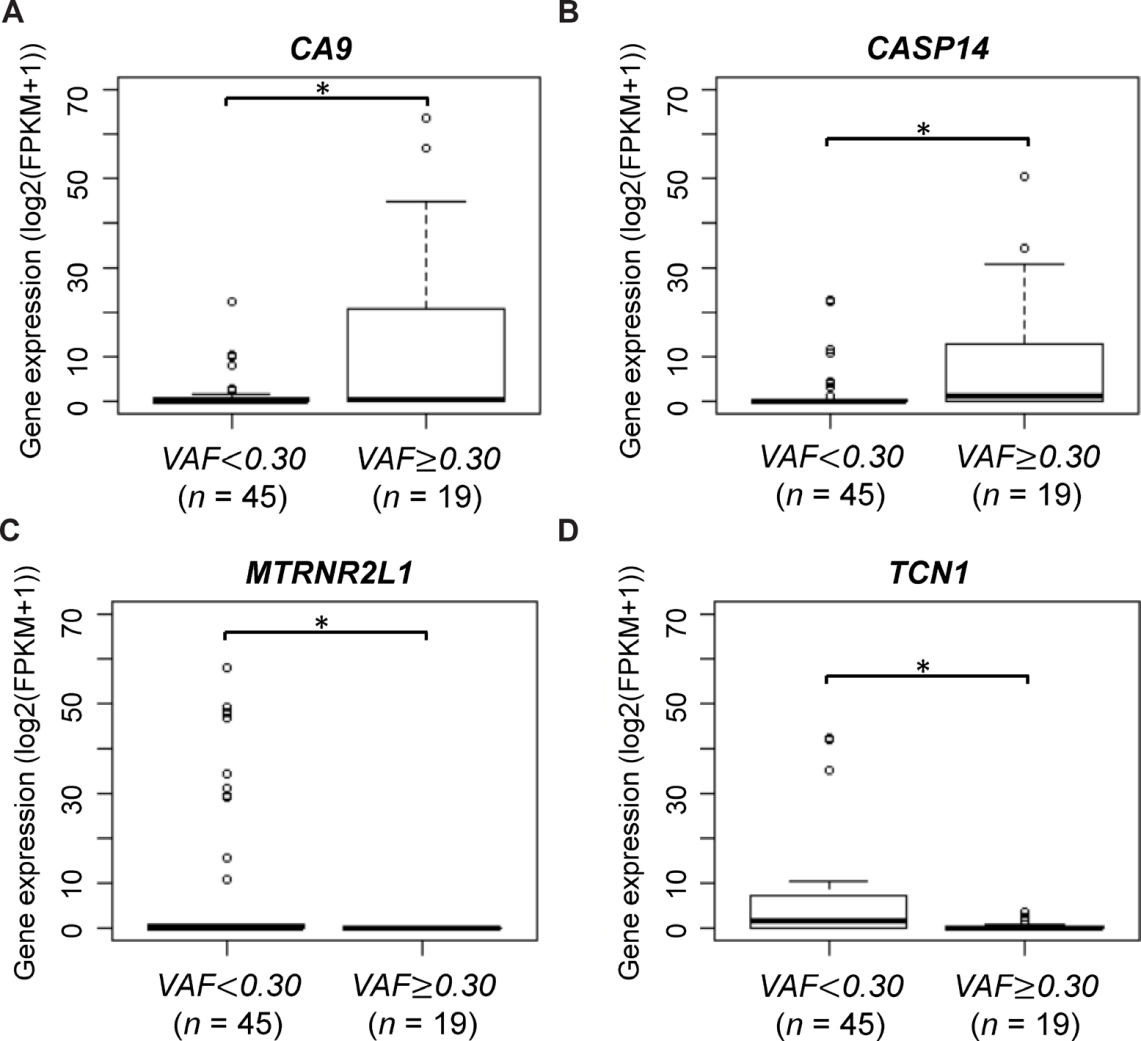
CA9 and MTRNR2L1 mRNA expression according to HMCN1 variant allele frequencies (VAFs) Samples were divided into two groups using a VAF cutoff of 0.30 (< 0.30, *n* = 45 and ≥ 0.30, *n* = 19). The asterisk indicates statistical significance.

To identify genes for which the expression levels were associated with the *HMCN1* VAF, we analyzed mRNA expression levels of all annotated genes. Among the annotated genes, only two significantly exhibited different expression in terms of the *HMCN1* VAF. A high *CA9* and *CASP14* expression level (*P* = 0.043 and 0.024, respectively) and low *MTRNR2L1* and *TCN1* expression level (*P* = 0.024 and 0.043, respectively) were found to be significantly associated with a higher VAF (Figure 3). *CA9* encodes carbonic anhydrase IX, an endogenous marker of hypoxic cells in breast cancers. *CASP14* encodes caspase14, which is one of the apoptosis-related cysteine peptidase. *MTRNR2L1* encodes human MT-RNR2-like 1, for which detailed functions remain unknown and *TCN1* encodes a member of the vitamin B12-binding protein family, named “transcobalamin 1”.

### Relationship between intratumor heterogeneity and *HMCN1* VAFs

To investigate intratumor heterogeneity in individuals with *HMCN1* mutations, we measured the number of subclones across each sample. We performed this analysis using the SciClone [16] with VAFs from somatic SNVs and copy number estimates. We compared between the higher and the lower *HMCN1* VAF groups in terms of the number of subclones by Fisher's exact test. The distributions of the number of subclones were not significantly different between the two groups (*P* = 0.347) (Supplementary Figure 4A).

There is another index for intratumor heterogeneity, mutant-allele tumor heterogeneity (MATH), which can be calculated from VAF distribution in a sample [27]. Previous studies have shown that higher MATH score was correlated with poor prognosis in head and neck squamous cell carcinoma and colon cancer [27, 28]. We compared between the higher and the lower *HMCN1* VAF groups in terms of MATH scores by Wilcoxon signed rank test. In the 19 samples with higher *HMCN1* VAF, the mean MATH was 35.014 (*SD* = 11.300). Meanwhile, in the 45 samples with lower *HMCN1* VAF, the mean MATH was 33.919 (*SD* = 10.557). No significant differences between the two groups were detected (*P* = 0.771) (Supplementary Figure 4B). These findings indicate that the prevalence of the mutations in *HMCN1* might not be involved in the status of intratumor heterogeneity.

### Associations of *HMCN1* with common driver genes

As *TP53* and *PIK3CA* mutations are among the most common genetic aberrations in breast cancers [23], we compared the VAFs of these two driver genes with those of *HMCN1*. Among the 64 samples harboring mutations in *HMCN1*, 22 and 23 also harbored mutations in *TP53* and *PIK3CA*, respectively, and five harbored mutations in both genes.

In the 22 samples with both *TP53* and *HMCN1* mutations, the mean *TP53* VAF was 0.697 (*SD* = 0.249) and the mean *HMCN1* VAF was 0.288 (*SD* = 0.201). Meanwhile, in the 23 samples with both *PIK3CA* and *HMCN1* mutations, the mean *PIK3CA* VAF was 0.442 (*SD* = 0.269) and the mean *HMCN1* VAF was 0.230 (*SD* = 0.148). A paired *t*-test showed that VAFs of the two driver genes were significantly higher than that of *HMCN1* (*TP53*; *P* < 0.01, *PIK3CA*; *P* < 0.01) (Supplementary Figure 5), indicating that mutations in *HMCN1* occurred later in the tumor evolutionary process than the mutations in *TP53* and *PIK3CA*. This finding suggests that the mutations in *HMCN1* might be involved in breast cancer progression.

### *HMCN1* mutations and clinical outcomes

**Figure 4:**
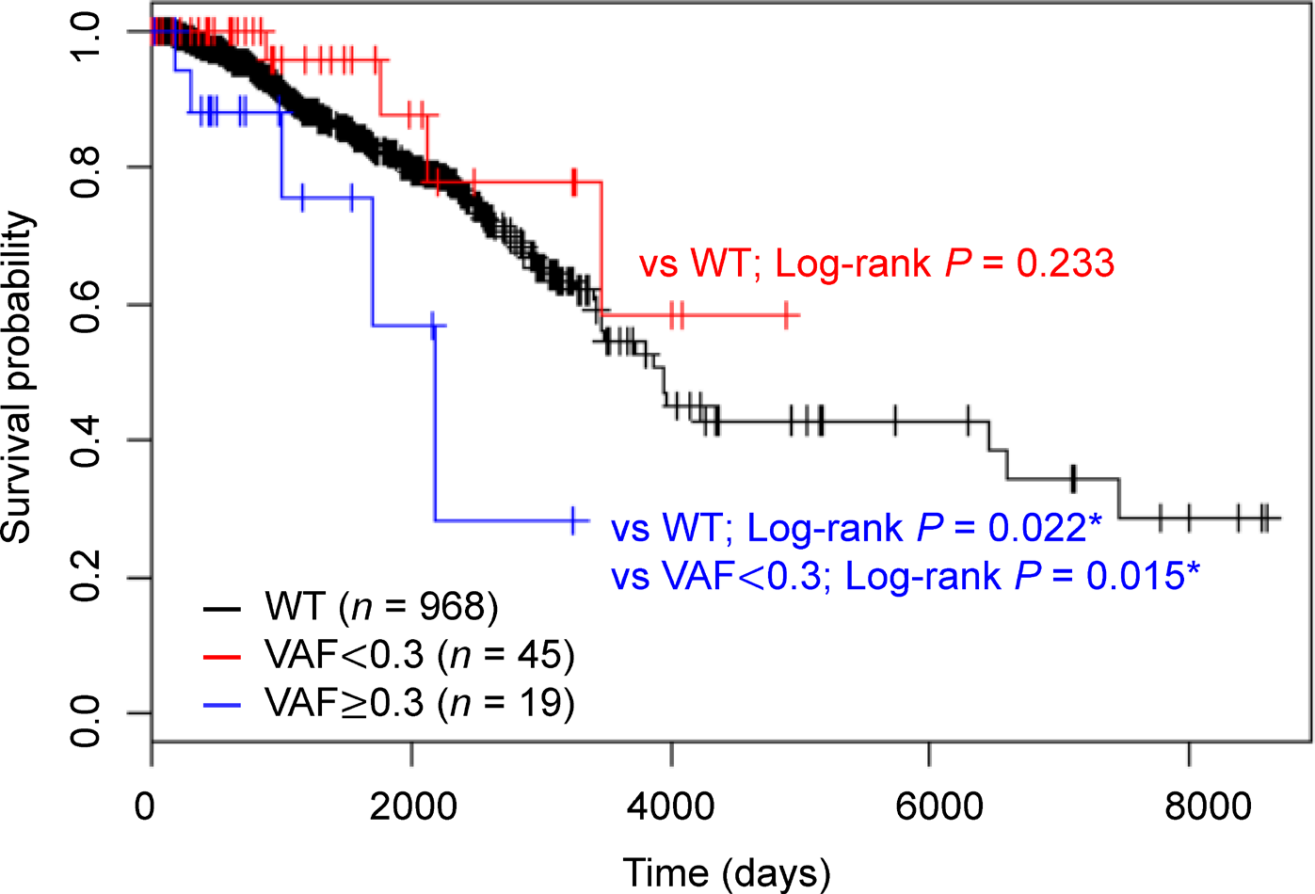
Kaplan–Meier analysis of overall survival according to HMCN1 variant allele frequencies (VAFs) Samples were divided into three groups using a VAF cutoff of 0.30 (< 0.30, red, *n* = 45 and ≥ 0.30, blue, *n* = 19) or WT (black, *n* = 968). The log-rank test was used to evaluate the statistical significance of the difference between the two survival curves (VAF of ≥ 0.30 vs. VAF of < 0.30, VAF of < 0.30 vs. WT and VAF of ≥ 0.30 vs WT).

Next, we evaluated the association between *HMCN1* mutation status and clinical variables by χ^2^ test or Fisher's exact test. We found tumor size (*P* = 0.028) and molecular subtype (*P* = 0.021) were related with the *HMCN1* mutation (Table 3). To assess the relationship of the *HMCN1* VAF with prognosis, the 64 samples harboring *HMCN1* mutations were divided into two groups according to VAFs and subjected to an OS analysis. These groups were also compared with individuals without *HMCN1* mutations (wild-type; WT). The resulting Kaplan–Meier plot shows that a higher *HMCN1* VAF significantly correlated with poor prognosis (log-rank test: vs. WT; *P* = 0.022 and vs. VAF of < 0.30; *P* = 0.015) (Figure 4). Concordantly, in a multivariate Cox proportional hazards regression analysis adjusted for the covariates of lymph node status, tumor grade, tumor size, and age, the VAF (*P* = 0.036) and lymph node status (*P* = 0.012) were significantly associated with poor prognosis (Table 4).

**Table 3:** Clinical data of 1,044 breast cancer patients

Variables		Overall	WT	MT	*P*-value^a^
		*n* = 1,044	*n* = 980	*n* = 64	
		No. (%)	No. (%)	No. (%)	
Lymh node status					0.175
	Negative	485 (46.5)	450 (45.9)	35 (54.7)	
	Positive	540 (51.7)	513 (52.3)	27 (42.2)	
	Unknown	19 (1.8)	17 (1.7)	2 (3.1)	
Tumor grade					0.080
	1	172 (16.5)	163 (16.6)	9 (14.1)	
	2	582 (55.7)	536 (54.7)	46 (71.9)	
	3	239 (22.9)	231 (23.6)	8 (12.5)	
	4	20 (1.9)	19 (1.9)	1 (1.6)	
	Unknown	31 (3.0)	31 (3.2)	0	
Tumor size (cm)					0.028^*^
	< 2	267 (25.6)	255 (26.0)	12 (18.8)	
	2–5	603 (57.8)	556 (56.7)	47 (73.4)	
	≧ 5	171 (16.4)	166 (16.9)	5 (7.8)	
	Unknown	3 (0.3)	3 (0.3)	0	
Molecular subtype					0.021^*^
	Luminal A	401 (38.4)	385 (39.3)	16 (25.0)	
	Luminal B	171 (16.4)	163 (16.6)	8 (12.5)	
	HER2-enriched	65 (6.2)	58 (5.9)	7 (10.9)	
	Basal-like	132 (12.6)	123 (12.6)	9 (14.1)	
	Normal	23 (2.2)	19 (1.9)	4 (6.3)	
	Unknown	252 (24.1)	232 (23.7)	20 (31.3)	
Age (year)					0.450
	Median (range)	59 (27–90)	59 (27–90)	59 (34–90)	
	< 50	276 (26.4)	261 (26.6)	15 (23.4)	
	≧ 50	743 (71.2)	649 (66.2)	49 (76.6)	
	Unknown	25 (2.4)	25 (2.6)	0 (0)	

Abbreviations: 95% CI, 95% confidence interval; MT; *HMCN1* mutant, VAF; variant allele frequency; WT, wild-type.

^a^Astarisk indicates statistical significance.

**Table 4:** Multivariate Cox proportional hazards regression analysis of overall survival according to the clinical characteristics of 64 breast cancer patients

Variables	Hazard Ratio (95% CI)	*P*-value^a^
Lymph node status		
Positive vs Negative	97.931 (2.709–3539.805)	0.012^*^
Tumor grade		
3–4 vs 1–2	9.468 (0.042–2147.487)	0.417
Tumor size, cm		
2–5 vs ≤ 2	1.281 (0.159–10.302)	0.816
> 5 vs ≤ 2	29.032 (0.284–2966.692)	0.154
Age		
≥ 50 vs < 50	0.114 (0.011–1.169)	0.068
VAF		
≥ 0.30 vs < 0.30	17.950 (1.216–264.976)	0.036^*^

Abbreviations: 95% CI, 95% confidence interval; VAF, variant allele frequency.

^a^Astarisk indicates statistical significance.

To exclude the possibility that this significant association is not attributable to bias in the impact of mutations in the two groups, we evaluated the impact of single nucleotide variants in *HMCN1* on protein structure and function using PolyPhen-2 scores [29]. The Pearson correlation coefficient between VAFs and PolyPhen-2 scores of *HMCN1* was −0.204 (*P* = 0.151), indicating no significant correlation. To analyze the relationship between prognosis and PolyPhen-2 scores, the 64 samples were divided into two groups using a PolyPhen-2 score of 0.85 as a cutoff (higher, *n* = 27 and lower, *n* = 24); this score ranges from 0 to 1 and yields predictions of “probably damaging” (> 0.85), “possibly damaging” (0.85–0.15), or “benign” (< 0.15). We found that the Polyphen-2 score of nonsynonymous *HMCN1* mutations did not significantly associate with breast cancer prognosis (PolyPhen-2 scores of < 0.85 vs. WT; *P* = 0.801 and PolyPhen-2 scores of ≥ 0.85 vs WT; *P* = 0.671) (Supplementary Figure 6).

These results suggest that the *HMCN1* VAF is an independent prognostic factor for OS, such that a higher VAF may be associated with poor survival in patients with breast cancer.

### Correlations with potential prognostic factors

**Figure 5:**
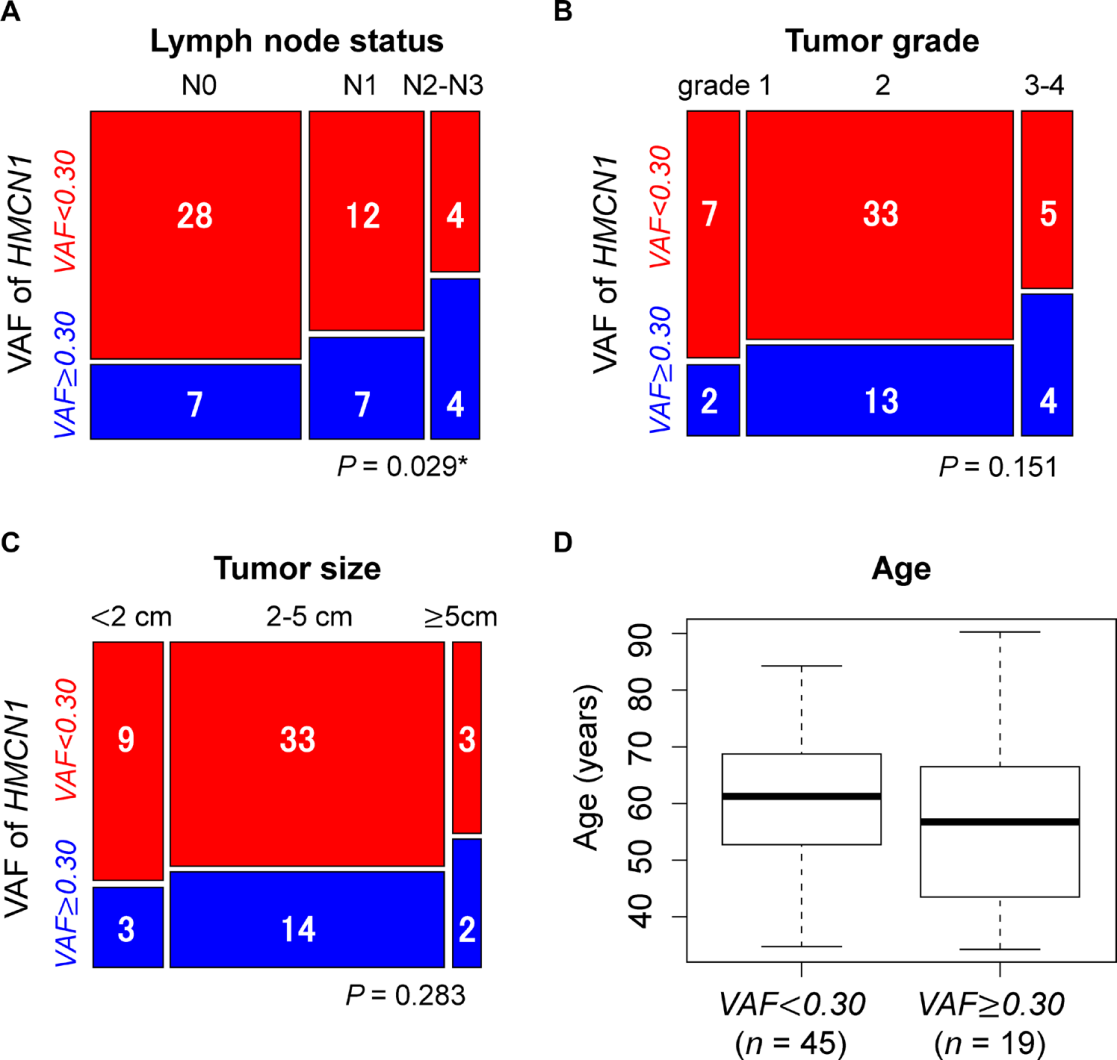
Associations of the *HMCN1* variant allele frequency (VAF) with clinical characteristics of (**A**) lymph node status, (**B**) tumor grade, (**C**) tumor size, and (**D**) patient age. Samples were divided into two groups using a VAF cutoff of 0.30 (< 0.30, red, *n* = 45 and ≥ 0.30, blue, *n* = 19). Blue and red squares in mosaic plots indicate sample counts from the higher and lower VAF groups, respectively. In the TCGA dataset, the lymph node status for two cases were not provided. The asterisk indicates statistical significance.

We next evaluated the association of the *HMCN1* VAF with individual clinical characteristics (lymph node status, tumor grade, tumor size, and age) in the 64 tumor samples, which were divided into three groups by lymph node status: N0, N1, and N2–N3. Samples were also divided into three groups by tumor grade: grades 1, 2, and 3–4. Regarding lymph node status, tumor grade, and tumor size, we examined whether a higher VAF was associated with significantly higher stages of clinical features using the Cochran–Armitage trend test. We found a significant association of a higher VAF with a much higher lymph node status (*P* = 0.029) (Figure 5A). By contrast, the tumor grade (*P* = 0.151) and tumor size (*P* = 0.283) were not significantly associated with the *HMCN1* VAF (Figure 5B and 5C). The mean ages of patients (*n* = 64) in the higher and lower VAF groups were 58.05 (*SD* = 17.95) years and 61.41 (*SD* = 11.85) years, respectively. A *t*-test revealed no significant difference in the mean age between the groups (*P* = 0.461) (Figure 5D).

We also used other 15 types of cancer dataset from TCGA and examined the association between *HMCN1* VAFs and OS. In the 15 types of cancer, only cervical squamous cell carcinoma and endocervical adenocarcinoma (CESC) samples showed significantly poorer OS in the samples with higher *HMCN1* VAFs (*n* = 7, VAF ≥ 0.30) than those with lower VAFs (*n* = 15, VAF < 0.30) (log-rank test: *P* = 0.048) (Supplementary Figure 7). Although, we applied a Cox proportional hazards regression analysis with the covariates of age, tumor grade, and *HMCN1* VAFs, the VAFs were not associated with poor prognosis (HR = 5.436, 95% CI: 0.543–54.432, *P* = 0.150).

## DISCUSSION

Breast cancers are known to exhibit a large degree of genetic heterogeneity. In this study, we analyzed intratumor heterogeneity using VAFs calculated from a set of breast cancer cases registered in TCGA. Through a VAF-based analysis of mutations, we showed that VAFs of *HMCN1* was possibly associated with breast cancer prognosis. Although the detailed function of *HMCN1* in humans remains unknown, mutations in *HMCN1* might be associated with cancer cell invasion and metastasis. Using the data of the DRIVE dataset, the CIMBA dataset, and the Foundation One dataset from breast cancer patients, we could not validate our result because the *HMCN1* VAF and survival time information could not be obtained. In cervical cancer from TCGA, however, survival time between the two groups of HMCN1 VAF values were also significantly different. Cervical cancer, like breast cancer, is known to associated with hormone estrogen. Therefore, this result may support the prognostic impact of HMCN1 on breast cancer. It will be possible to further evaluate the validity of our results by accumulating more cohorts.

*HMCN1* encodes a large extracellular protein belonging to the immunoglobulin superfamily and comprises several distinct domains, including the von Willebrand factor and Ig-like C2-type domains [30]. *HMCN1* mutations are believed to correlate with age-related macular degeneration [31]. According to another recent report, *HMCN1* acts as a suppressor of gallbladder cancer metastasis [32] and is commonly mutated in certain samples of head and neck squamous cell carcinoma [33]. There are at least three possible functional implications of the mutations in *HMCN1* in breast cancer metastasis. First, *HMCN1* is also known as *FBLN6* (fibulin 6) and one of the extra cellular matrix (ECM) proteins [34, 35]. The fibulins are shown to be involved in basement membrane and formation of stable cell-to-cell interactions, leading to organization and stabilization to ECM structure [36]. When HMCN1 does not function properly in cancer cell, sufficient cell adhesion might be inhibited and as a result of promoting cancer invasion due to instability of HMCN1 caused by the variants in the gene. For example, previous study reported that epigenetically silenced fibulin 5 promotes invasion and metastasis in lung cancer [37]. Second, *HMCN1*, which contains estrogen receptor binding site, seems to be associated with postpartum depression symptoms [38], and one of its functions is suggested to be cell adhesion [39]. Therefore, HMCN1 mutations may be associated to cancer proliferation and metastasis because of the disruption of these functions. Finally, other studies have shown that HMCN1 might interact with DDX1 [40], a DEAD box protein with RNA helicase activity [41, 42]. Notably, the expression of *DDX1* was reported to decrease under hypoxic conditions [43], and intratumor hypoxia is associated with cancer metastasis and, consequently, patient mortality [44, 45]. Indeed, an earlier report found that *DDX1* correlated with ovarian tumor metastasis and progression [46]. In the current study, we found that *CA9*, the expression of which is also associated with tumor hypoxia [43, 47], was expressed at significantly higher levels in patients with higher *HMCN1* VAFs than in those with lower VAFs. Therefore, the *HMCN1* VAF may indicate the metastatic potential of a breast cancer.

Although metastasis is the main cause of death among breast cancer patients, factors involved in metastasis remain poorly characterized. It is more difficult to identify genetic factors associated with metastasis, a complex process, than to identify driver genes [48]. Differences in genetic heterogeneity between metastatic and primary tumors may affect treatment efficacy and thus represent one of the biggest obstacles toward cure for breast cancer. Using VAFs to explicitly address heterogeneity, we successfully identified *HMCN1* as a possible metastatic factor. Although further experimental validation is needed to determine the involvement of *HMCN1* in metastasis, this approach could be used to screen genes that have not previously been investigated.

In this study, we focused on nonsynonymous mutations and indels. However, intratumor heterogeneity may also be caused by copy number variants or mutations in noncoding regions, such as those in cis-regulatory elements and splice sites [49–52]. Moreover, epigenetic alterations can also promote cancer progression [53]. As genome-wide epigenetic datasets from normal cells grow rapidly [54], epigenome data analyses of cancer cells will allow evaluations of the impacts of epigenetic factors on intratumor heterogeneity.

In conclusion, to our knowledge, this is the first study to identify *HMCN1* as a potential metastatic factor in breast cancer using a comparative analysis of genomic and transcriptomic data registered in TCGA. In addition to the standard classification of breast tumors based on the four molecular types, the use of VAFs, which reflect tumor evolution, might provide further genetic profile information that can be used to characterize tumor samples. Our approach allows us to identify new diagnostic markers or candidate genes for targeted therapy and is therefore expected to facilitate precision medicine.

## MATERIALS AND METHODS

### Datasets

A total of 1,080 RNA-seq and variant datasets from breast cancers were downloaded from TCGA (https://portal.gdc.cancer.gov/). For variant data, we used VCF files generated by comparing matched tumor–normal pairs using the Mutect2 software package. We also downloaded the associated clinical patient data. Similarly, dataset from other 15 types of cancer were obtained from TCGA: Bladder urothelial carcinoma (BLCA; *n* = 416), cervical squamous cell carcinoma and endocervical adenocarcinoma (CESC; *n* = 307), colon adenocarcinoma (COAD; *n* = 605), glioblastoma multiforme (GBM; *n* = 938), head and neck squamous cell carcinoma (HNSC; *n* = 512), kidney renal clear cell carcinoma (KIRC; *n* = 697), lower grade glioma (LGG; *n* = 938), liver hepatocellular carcinoma (LIHC; *n* = 378), lung adenocarcinoma (LUAD; *n* = 587), lung squamous cell carcinoma (LUSC; *n* = 503), ovarian serous cystadenocarcinoma (OV; *n* = 443), prostate adenocarcinoma (PRAD; *n* = 503), skin cutaneous melanoma (SKCM; *n* = 472), thyroid carcinoma (THCA; *n* = 504), and uterine corpus endometrial carcinoma (UCEC; *n* = 604).

### Mutation analysis

In this study, we only considered mutations with a coverage depth of ≥ 20. We extracted gene mutations [i.e., nonsynonymous substitutions (missense and nonsense mutations) and indels (frameshift insertions and frameshift deletions)] observed in ≥ 50 samples. We used this cutoff because the lower limit of the average mutation rate for significantly mutated genes was approximately 2–4% [23]. VAFs were calculated as the proportion of variant allele reads to total reads at the mutation site. When a sample harbored multiple mutations in the same gene, the larger VAF was used as the VAF for the gene. The VAF was adjusted for tumor purity estimate. This estimate, which was derived from immunohistochemistry analysis, was downloaded from the previous study [25].

The number of subclones in a tumor cell were inferred by both VCF files and DNA copy number variation data using the R package SciClone with default settings [16].

### Statistical analysis

Statistical analysis was conducted using the R software, version 3.3.1 (R Project for Statistical Computing, Vienna, Austria), and JMP Pro, version 13.0 (SAS Institute Inc., Cary, NC, USA). A χ^2^ test or Fisher's exact test (when ≥ 1 cells had an expected frequency of ≤ 5 in any clinical group) was used to evaluate the relationships between the mutation status and clinical variables. We also used the test for comparison the number of subclones. OS was estimated using the Kaplan–Meier method in the R survival package (version 2.41–3). For the multivariate analysis, adjusted hazard ratios (HRs) with 95% confidence intervals (95% CIs) were calculated using a Cox proportional hazards regression model. We used edgeR (version 3.16.5), a Bioconductor package (http://www.bioconductor.org/packages/release/bioc/html/edgeR.html), to detect genes differentially expressed between two groups. For each gene, the R exactRankTests package (version 0.8–28) was used to evaluate the difference in both expression levels and MATH between groups of samples. For categorical data such as tumor grade, tumor size category, and lymph node status, we used a one-sided Cochran–Armitage trend test to evaluate the existence of a linear relationship in terms of VAFs. We used Welch's *t*-test to compare continuous data between the two groups. An analysis of variance (ANOVA) model was used to compare the mean values of more than two groups. *P*-values were considered statistically significant at < 0.05 (^*^P < 0.05, ^**^P < 0.01).

## SUPPLEMENTARY MATERIALS FIGURES


