# Supplementary material for: Intratumor heterogeneity of HMCN1 mutant alleles associated with poor prognosis in patients with breast cancer
Source: Oncotarget. 2018 Sep 7;9(70):33337–47. doi: 10.18632/oncotarget.26071 (PMC6161790; doi:10.18632/oncotarget.26071)
Supplement: Supplementary file 1 [file oncotarget-09-33337-s001.pdf]

Intratumor heterogeneity of *HMCN1* mutant alleles associated with poor prognosis in patients with breast cancer

SUPPLEMENTARY MATERIALS

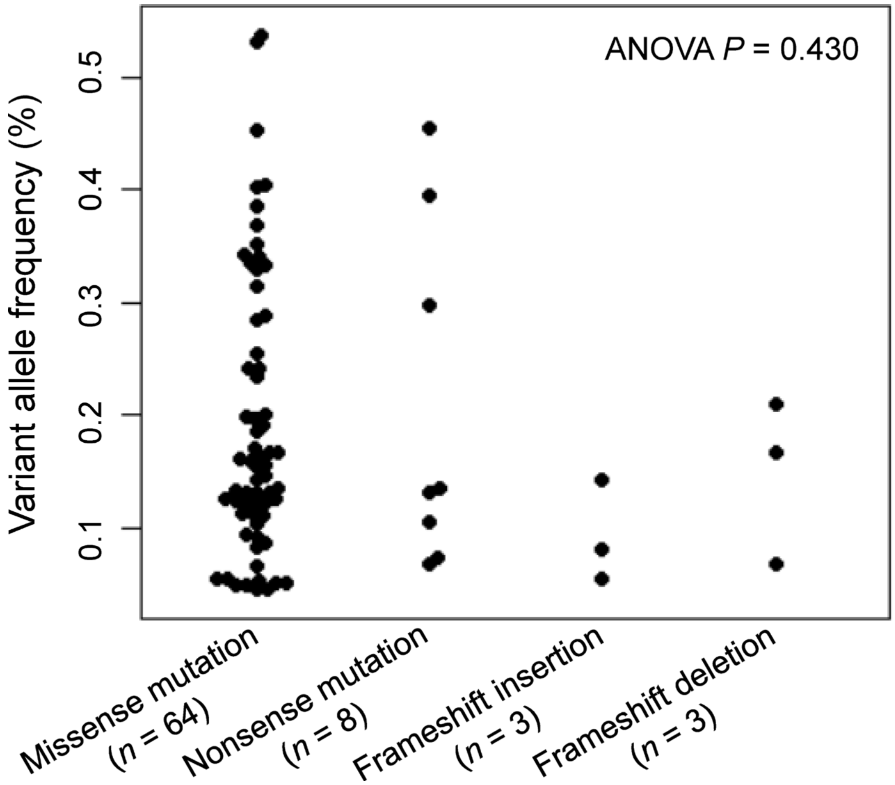

Supplementary Figure 1: Variant allele frequencies of each of the four types of mutations in *HMCN1*. The y-axis indicates variant allele frequency. Sixty-four missense mutations, eight nonsense mutations, three frameshift insertions, and three frameshift deletions were observed in *HMCN1*.

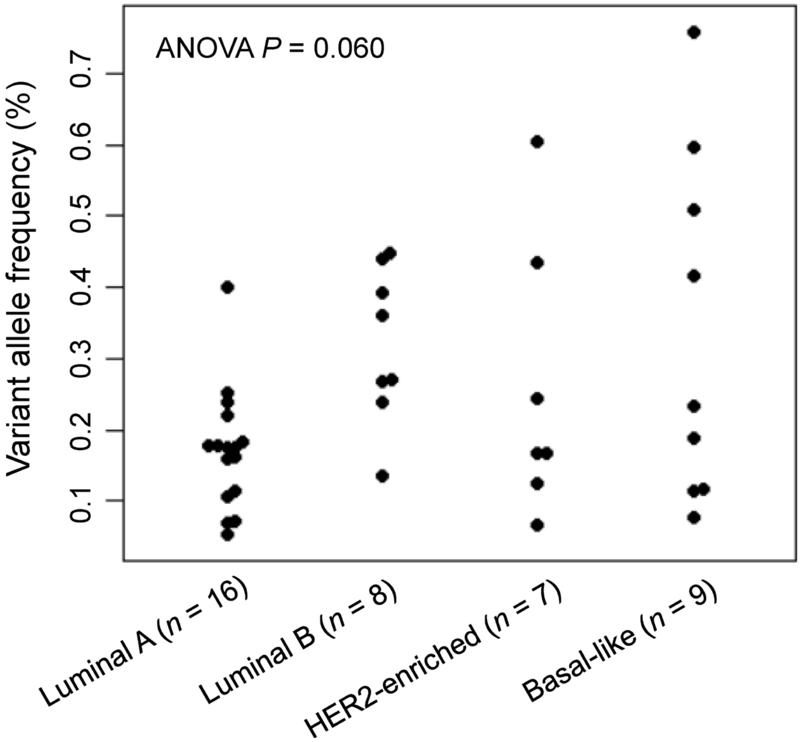

Supplementary Figure 2: *HMCN1* variant allele frequencies in each of the four molecular subtypes of breast cancer. The y-axis indicates the variant allele frequency. The 64 samples with *HMCN1* mutations were classified as follows: luminal A, 16; luminal B, eight; HER2-enriched, seven; basal-like, nine; and unknown, 24.

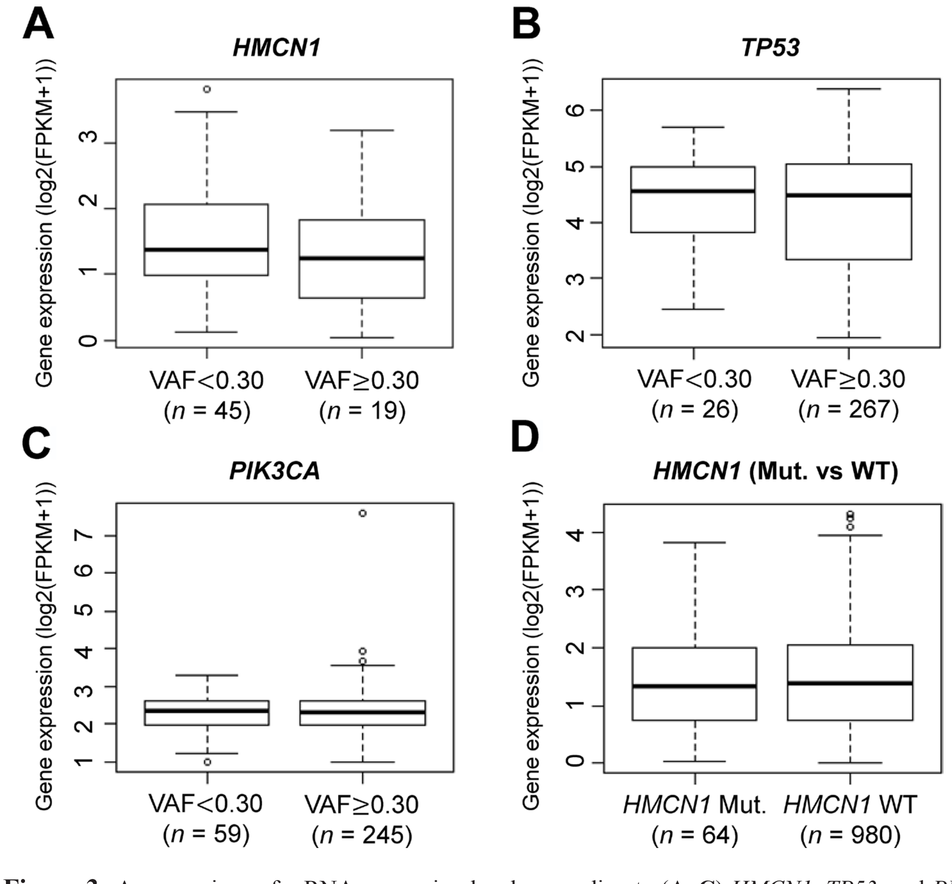

Supplementary Figure 3: A comparison of mRNA expression levels according to (**A–C**) *HMCN1*, *TP53*, and *PIK3CA* variant allele frequencies and (**D**) *HMCN1* mutations. The asterisk indicates statistical significance ($P < 0.05$).

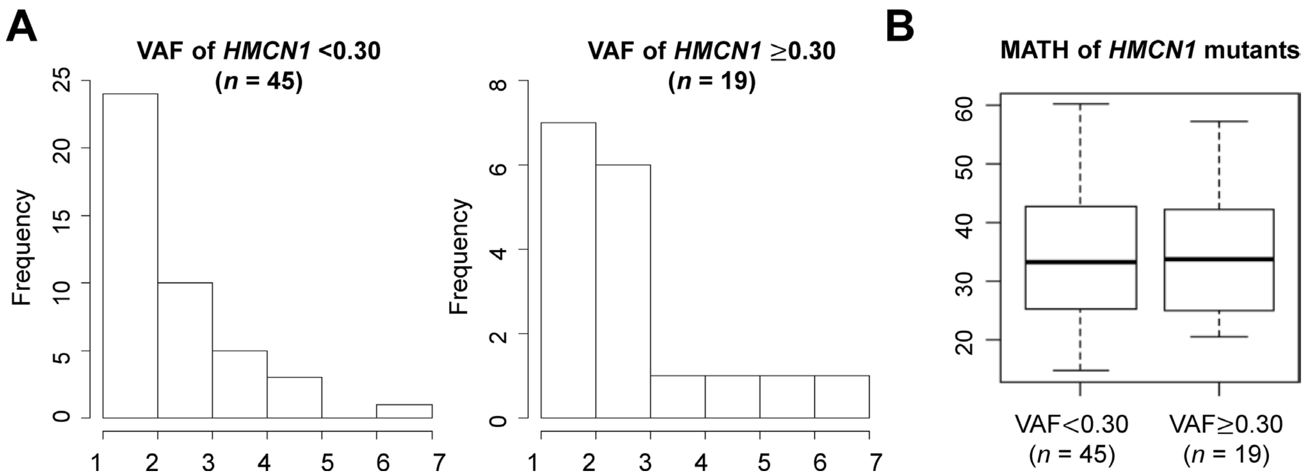

Supplementary Figure 4: Associations of the *HMCN1* VAF with number of subclones (**A**) and MATH (**B**). The asterisk indicates statistical significance ($P < 0.05$).

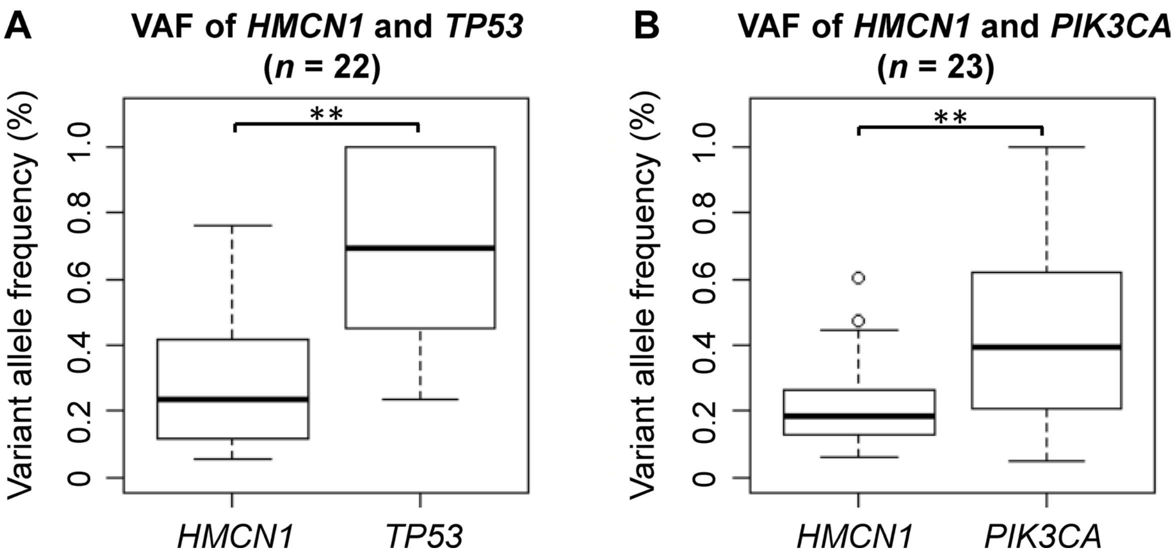

Supplementary Figure 5: A comparison of *HMCN1* variant allele frequencies (VAFs) with those of *TP53* and *PIK3CA*. (**A**) Comparison between VAFs of *HMCN1* and *TP53* ($n = 22$) and (**B**) between those of *HMCN1* and *PIK3CA* ($n = 23$). The asterisk indicates statistical significance.

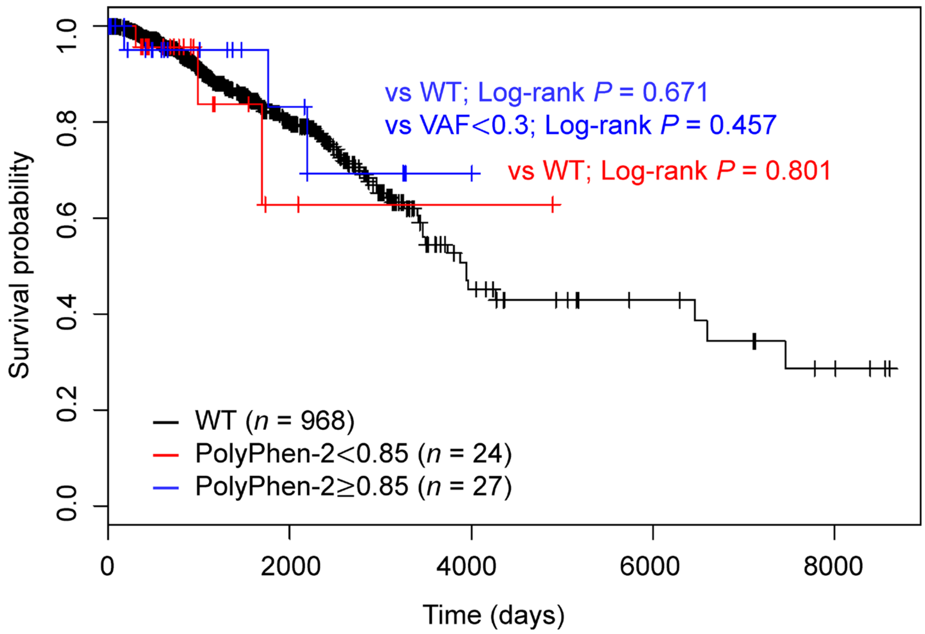

Supplementary Figure 6: A Kaplan–Meier analysis of overall survival according to the PolyPhen-2 scores of *HMCN1* mutations. Samples were divided into three groups using a PolyPhen-2 scores cutoff of 0.85 (< 0.85, red, $n = 24$ and ≥ 0.85, blue, $n = 27$) or WT (black, $n = 968$). The log-rank test was used to evaluate the statistical significance of the difference between the two survival curves.

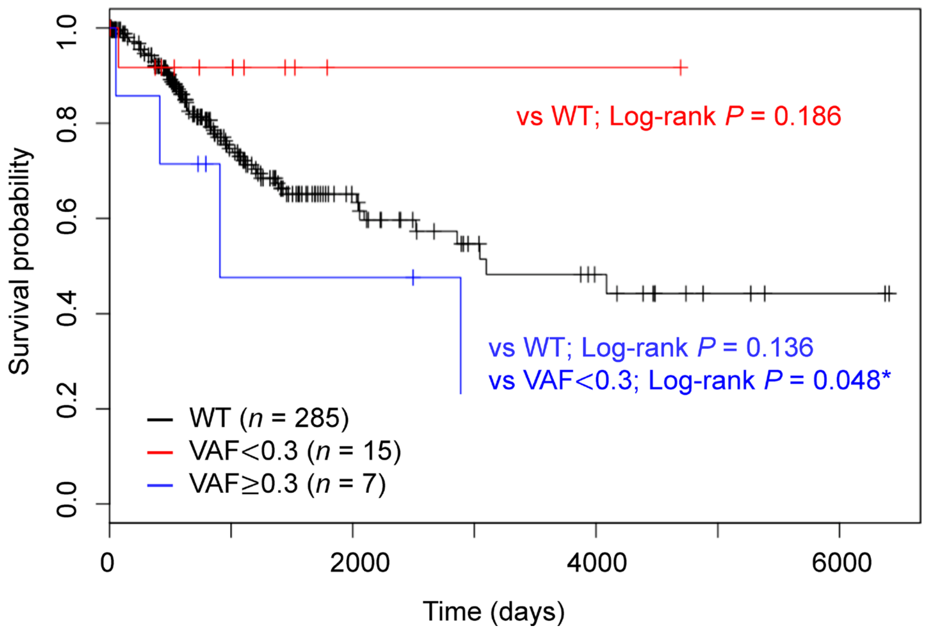

Supplementary Figure 7: A Kaplan–Meier analysis of overall survival according to *HMCN1* VAFs in CESC. Samples were divided into three groups using a VAF cutoff of 0.30 (< 0.30, red, $n = 15$ and ≥ 0.30, blue, $n = 7$) or WT (black, $n = 285$). The log-rank test was used to evaluate the statistical significance of the difference between the two survival curves (VAF of ≥ 0.30 vs. VAF of < 0.30, VAF of < 0.30 vs. WT and VAF of ≥ 0.30 vs WT).
